# Early nutritional management of cystic fibrosis infants born with meconium ileus

**DOI:** 10.1002/jpr3.70254

**Published:** 2026-10-15

**Authors:** Laetitia Mouly, Tom Ruffles, Chris Smith

**Affiliations:** ^1^ Brighton and Sussex Medical School University of Brighton and University of Sussex Brighton UK; ^2^ Royal Alexandra Children's Hospital University Hospitals Sussex NHS Foundation Trust Brighton UK; ^3^ Present address: St Helier Hospital Epsom and St Helier University Hospitals NHS Trust Sutton UK

**Keywords:** enteral feeds, infancy, intra‐operative nutrition, multidisciplinary work, total parenteral nutrition

## Abstract

Meconium ileus (MI) is a well‐recognised neonatal presentation of cystic fibrosis (CF) and is associated with a high nutritional risk. While nutritional management principles are outlined in international guidelines, there is limited literature describing the practical experiences of nutritional management. The aim of this communication was to describe nutritional factors observed during the first year of life in a series of MI‐CF infants. Data on nine surgically managed infants were reviewed from a UK CF centre. Timelines of (i) nutritional and surgical management and (ii) growth and intake outcomes are described. Findings highlight the heterogeneity of nutritional aspects in this group and the high prevalence of acute and chronic growth failure, further supporting the requirement for close nutritional monitoring. CFTR modulator therapies have revolutionised CF management and are increasingly available at younger ages. However, as treatment is initiated well after the neonatal period and acute MI, optimising nutritional management of MI‐CF infants remains a priority.

## Abstract (highlights)

**What is Known**

- Meconium ileus (MI) in cystic fibrosis (CF) is a high‐risk factor for poor nutritional and growth outcomes.
- International guidelines outline broad nutritional management for MI‐CF infants, but descriptions of practical experiences are lacking in the literature.

**What is New**

- There is wide heterogeneity in nutritional practical experiences for MI‐CF infants.
- In this cohort, breastfeeding rates were notably inferior to the average rates in CF populations.
- Despite the increasing access and use of CFTR modulator treatments, these are currently initiated well after the neonatal period and acute MI and therefore optimising nutritional management remains a clinical priority.

## 1 INTRODUCTION

Cystic fibrosis (CF) is a common life‐limiting autosomal recessively inherited condition. It is caused by mutations of the gene encoding the CF transmembrane conductance regulator. Optimisation and management of nutrition and nutritional status is a cornerstone to care, and a key priority starting immediately from diagnosis and continuing throughout life. This focus has evolved through strong and well‐established links that exist between nutritional status and acute and chronic health outcomes.^1^

Approximately 10%–20% of infants with CF (iwCF) will present with meconium ileus (MI) which is considered to be a neonatal emergency.^2^, ^3^, ^4^ In MI, thickened meconium blocks the small intestine which can lead to bowel obstruction, necrosis and perforation.^2^ Around 70% of MI patients require surgical management including bowel resection, stoma creation and closure.^1^ Recent European nutrition CF guidelines recommend total parenteral nutrition (TPN) may be required in the first months of life to support optimal growth.^1^, ^2^, ^5^ Alongside TPN, enteral nutrition (EN) must be initiated as soon as the bowel is unobstructed and progressed as tolerated by the infant.^1^, ^2^ It is widely recognised that infants with MI are at risk of poor nutritional outcomes^6^, ^7^, ^8^, ^9^ requiring proactive nutritional support. This is important as poor nutritional outcomes are associated with worsened growth trajectories, lung function and increased dependency on enteral feeds during childhood.^6^ The pursuit of optimal nutrition in early life has been identified as a key priority for all infants with CF but particular focus in MI infants is warranted,^1^, ^10^ especially with the paucity of the literature in this area.

Whilst the principles of nutritional management of MI in iwCF has been described,^1^, ^2^ details on nutritional factors and practical experience are lacking.^2^, ^4^, ^11^ The primary aim of this short communication is to describe the practical nutritional timeline in a sample of iwCF who had surgically treated MI. The timeline focuses on (i) nutritional and surgical management and (ii) intake and growth outcomes.

## 2 METHODS

### 2.1 Ethics statement

Following review by the Brighton and Sussex Medical School Individual Research Project research ethics process, the project was classified as a retrospective clinical audit and formal ethics application was not required.

### 2.2 Sample

A database of CF patients managed by the Brighton UK CF Centre was used to identify iwCF born between 2011 and 2021 with MI. Those with MI requiring surgical treatment were included and those treated conservatively were excluded.

Health records were accessed between October 2022 and December 2023. Patients born between 2011 and 2022 were selected for inclusion. There was a similar spread of patients across this timeframe. Indeed, 45% of patients were born between 2011 and 2016, 55% were born between 2016 and 2022.

### 2.3 Nutritional and surgical management

Patients’ records were accessed for data collection. Details collected included date of: (i) Bowel resection (stoma creation) and stoma closure, (ii) Start and stop of TPN after each surgery and (iii) Start and stop of EN after each surgery.

### 2.4 Growth and intake

Anthropometrics and mode of feeding was collected at five time points: birth, 3, 6, 9, and 12 months (± 6 weeks). Centiles and *Z*‐scores were calculated using the World Health Organisation Royal College of Paediatrics and Child Heath application. Growth failure (GF) was defined as a fall across 1 or more weight centile spaces, if birthweight was below the 9th centile or a fall across two or more weight centile spaces, if birthweight was between the 9th and 91st centiles.^12^

### 2.5 Statistical analysis

Data were analysed descriptively using Microsoft Excel. Categorical variables are presented as frequencies and percentages, and discrete variables as days or weeks as appropriate.

## 3 RESULTS

Nine cases were identified of surgically managed MI from 2011 to 2021.

### 3.1 Nutritional and surgical management

The nutritional management timeline of this sample is described in Table 1.

**Table 1:** Description of nutritional journey of nine iwCF MI patients around stoma formation and closure.

Basic demographics	Patient 1	Patient 2	Patient 3	Patient 4	Patient 5	Patient 6	Patient 7	Patient 8	Patient 9
Sex	M	F	F	M	F	M	F	M	M
Genotype	ΔF508	ΔF508	ΔF508	ΔF508	ΔF508	ΔF508	ΔF508	ΔF508	ΔF508
	ΔF508	ΔF508	2732insA	ΔF508	R553X	C.3528 delC	ΔF508	ΔF508	ΔF508
Gestation (weeks + day)	38	40 + 2	35 + 2	40	40	39 + 5	37	40	40 + 3
Weight *Z*‐score									
At Birth	−0.319	−0.618	1.144	−0.231	−0.411	−0.708	0.386	0.618	0.854
At 1 year	0.985	−0.479	0.673	0.829	−0.239	0.465	−0.071	1.036	1.036
Events (day of life)									
Stoma formation									
Stoma formation	4	2	4	2	1, 21, 36^a^	1	0	1	2
Initiation of TPN	4	4	6	4	1	1	0	4	2
Introduction of EN	5	10	7	27	6, 27, 39^a^	6	6	4	5,42
Cessation of TPN (if before closure)	20	Continued	10	Continued	52	33	Continued	10	17, 44, 53, 81^b^
Initiation of TPN again	n/a	n/a	n/a	n/a	56	n/a	n/a	16	38, 45, 62^b^
Stoma closure									
Stoma closure	90	52	85	73	88	112	36	34	82
Initiation of TPN	91	n/r	85	n/r	n/r	113	n/r	n/r	82
Introduction of EN	102	55	89	77	91	120	44	46	85
Cessation of TPN	102	66	89	77	101	124	44	46	91
Initiation of TPN	n/r	n/r	n/r	81	n/r	n/r	n/r	n/r	n/r
Cessation of TPN				147					

Abbreviations: EN, enteral nutrition; F, Female; iwCF, infants with cystic fibrosis; M, Male; MI, meconium ileus; n/a, not applicable; n/r, not required; TPN, total parental nutrition.

^a^ Patient 5 had one bowel washout on day 1, and two bowel resections on day 21 and 36, therefore they were introduced to enteral feed on three separate occasions.

^b^ Patient 9 started and stopped TPN a total of five times due to their high stoma output, followed by line infections and clot complications.

The mean duration of stoma bag use was 68.2 days (range from 33 to 111).

The mean length of TPN use was 56.5 days (range from 8 to 139).

The mean time before EN was initiated after stoma formation was 7 days (range 1 to 25).

The mean time before EN was initiated after stoma closure was 6.3 days (range 3 to 12).

### 3.2 Growth and intake

Figure 1 illustrates the growth pattern of our cohort across their first year of life.

**Figure 1:**
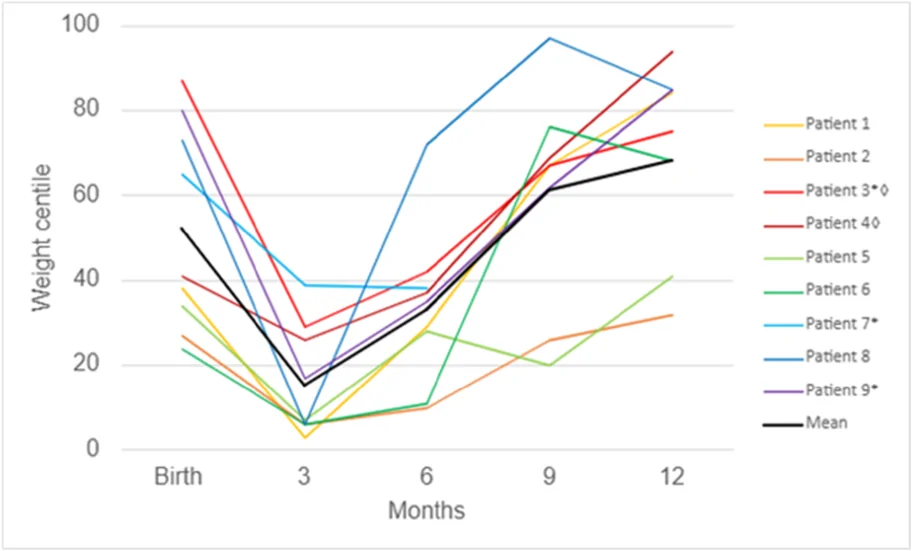
Weight centiles for 9 iwCF MI patients over the first year of life. *Did not achieve birth centile at 1 year. ◊ Experienced growth failure. iwCF, infants with cystic fibrosis; MI, meconium ileus.

Breastfeeding (BF) decreased from 89% at birth to 33% at 3 months, plateaued for 6 months, and fell to 22% and 11% by 9 and 12 months.

Over the first year of life, the average BF rate was of 38%. All nine patients had their feeding regimen collected at five different time intervals. Across 45 feeding regimen collected, 17 included BF (either exclusively or with supplemental formula).

No patient was exclusively BF during their first year of life.

All patients received pancreatic exocrine replacement therapy (PERT) based on elastase results, genotype and clinical picture.

## 4 DISCUSSION

We report a single centre's 10‐year experience of nutritional management in iwCF presenting with MI who were surgically managed. While we report on a small number of infants, this is an important contribution given the paucity of literature describing the practical experiences of nutritional management in MI CF infants and therefore descriptions of care are important.

All patients received input from the CF multi‐disciplinary team, with TPN and EN initiated post‐surgery and close monitoring according to international guidelines.^1^, ^10^ Despite this, we observed wide variation in nutritional support timing, growth outcomes, and intake, reflecting the complexity and diversity of nutritional care. This is partially explained by complications including complex management of high stoma outputs and line infections. Although data are limited, EN was initiated at average 8 days post‐operatively, comparable to 9 days in a Polish cohort of 10 MI‐CF patients.^13^ However, the focus of this study was the surgical management of MI. Notably, our cohort had earlier stoma closure (between 34 and 112 days, mean 72.4 days) whereas the Polish cohort had their stoma closure later on (between 12 and 30 months, mean 19.4). As such, the study only briefly comments on the initiation of EN after stoma formation. Both the Polish and our case series had early stoma formation during the first days of life, which may explain the similarity in EN initiation timeframe. Our case series complements these findings as we describe the dynamic relationship between TPN and EN, which is an essential factor for successful stoma formation and closure. Moreover, the variability in nutritional support observed in our cohort aligns with the variability in other aspects of MI care such as surgical management. Indeed, the polish cohort had individualised surgical management of MI in each infant.^10^ The variability in care for iwCF born with MI exists even within each county, as seen in the United Kingdom.^4^ This highlights the need for collaborative multi‐centre studies collecting data on both surgical and nutritional outcomes of MI‐CF infants to identify factors contributing to these variabilities.

The complexity of MI‐CF care underscores the need for close MDT input from paediatric surgeons, neonatologist, and dieticians^1^ in the management of these patients.

The BF rate in our group was 38%, notably inferior to the average of 50%–60% reported in the CF population,^14^ although specific rates in the context of MI are lacking. Exclusive BF is controversial in iwCF, as recent studies suggest it may be associated with sub‐optimal growth,^15^ an issue of acute concerns in MI patients. However, guidelines^1^ continue to support BF and these findings support the need for targeted support in this group.

GF was seen in 7/9 (77%) iwCF. This is higher than the literature's finding of 40% in CF‐MI patients.^5^, ^6^ Of note, all seven patients with GF were breastfed at the time of MI but only three continued BF until 6 months of age. Stress and decreased latching time are key factors that negatively impact breast milk production,^16^ of which mothers experience during hospital stay. This underscores the fragility of BF in this group and the increased focus required to support and optimise it.

Three iwCF failed to achieve their birth centiles by 1 year of age. Several factors likely contributed to this including *Pseudomonas aeruginosa* infection (patient 7) and significant surgical complications (patient 9). Growth outcomes and particularly weight at 1 year of life is a predictive factor for later respiratory function for CF infants.^17^ MI is a poor prognostic factor for growth and respiratory outcomes, highlighting the additional support CF‐MI patients require.^6^

All infants were initiated on PERT. However, data on sodium supplementation and PERT dosing were not collected. As both can influence growth and optimise feeding in MI, we acknowledge this point as a limitation of our case series.

CFTR modulators (CFTRm) have revolutionised CF care, management and outcomes in the recent decades. Fundamental nutritional principles have, as a result, also undergone significant re‐evaluation but the focus on optimisation of iwCF nutritional care has remained unchanged. Whilst the age of license of CFTRm is reducing, there is currently no license for use invitro.^18^ Emerging cases have been reported where CFTRm were administered during pregnancy to mothers carriers of iwCF, resolving MI in utero. These were initially encouraging but are now nuanced with unsuccessful cases where infants still required emergency surgery after delivery.^19^, ^20^ Nevertheless, CFTRm may limit the need of invasive surgeries for iwCF born with MI. This could lead to less TPN use and earlier introduction of EN, improving gut function earlier on. Ultimately, more evidence needs to be collected to differentiate the impact of CFTRm use versus the natural history of MI in iwCF.^19^, ^20^ As CFTRm become more readily available, systematically collecting evidence on the nutritional timeline and outcomes for MI‐CF infants can only improve our understanding of the natural history and management of this presentation.

Our case series highlight a small number of iwCF who had surgically treated MI in one single centre in the United Kingdom. Given the variability of nutritional and growth outcomes observed, a larger multi‐centre sample size is required to identify key factors explaining this variability. Further documented nutritional practical experiences of MI‐CF infants can support standardised care for this complex patient group, which ultimately remains a research priority for the CF community.

## 5 CONCLUSION

MI presents a unique and challenging nutritional situation. Preventing GF, optimising nutritional status, and supporting BF remain key priorities as for all iwCF. However, in MI these must be considered alongside surgical interventions, physiologically altered bowels, hospitalisation and fluctuating nutritional routes.

## CONFLICT OF INTEREST STATEMENT

The authors declare no conflicts of interest.

## Data Availability

The authors have nothing to report.
